# ASSESSING THE IMPACT OF COVID-19 ON THE HEALTH AND WELLNESS OF THE LONG-TERM CARE WORKFORCE IN RURAL AND NORTHERN AREAS

**DOI:** 10.1093/geroni/igac059.374

**Published:** 2022-12-20

**Authors:** Shannon Freeman, Davina Banner, Hui Jun Chew, Tammy Klassen-Ross, Piper Jackson

**Affiliations:** University of Northern British Columbia, Prince George, British Columbia, Canada; University of Northern British Columbia, Prince George, British Columbia, Canada; University of Northern British Columbia, Prince George, British Columbia, Canada; University of Northern British Columbia, Prince George, British Columbia, Canada; Thompson Rivers University, Kamloops, British Columbia, Canada

## Abstract

There is growing recognition that the mental health and wellbeing of the LTCF workforce have been disproportionately impacted by COVID-19. Therefore, we sought to describe the experiences and challenges LTCF employees faced during COVID-19 in rural and northern communities and highlight factors affecting their ability to be resilient and provide high quality care. We conducted 53 qualitative interviews using zoom with LTCF care providers (care aides, nurses, social workers), staff (food service workers, recreation providers), and management between November 2021 and February 2021. Data was transcribed and thematic analysis undertaken. We will describe participants experiences stratified by LTCF employee type and highlight similarities and differences in participants experiences across geography and facility type (freestanding vs. co-located in hospital) and describe factors affecting well-being, job satisfaction, and retention. We will share an inventory of programs and strategies participants found useful to mitigate negative effects on their mental health and well-being.

